# Impact of familiarity with the format of the exam on performance in the OSCE of undergraduate medical students – an interventional study

**DOI:** 10.1186/s12909-024-05091-0

**Published:** 2024-02-23

**Authors:** Hannes Neuwirt, Iris E. Eder, Philipp Gauckler, Lena Horvath, Stefan Koeck, Maria Noflatscher, Benedikt Schaefer, Anja Simeon, Verena Petzer, Wolfgang M. Prodinger, Christoph Berendonk

**Affiliations:** 1 Department of Internal Medicine IV – Nephrology and Hypertension, Medical University of Innsbruck, Anichstrasse 35, 6020 Innsbruck, Austria; 2 Department of Urology, Medical University of Innsbruck, Anichstrasse 35, 6020 Innsbruck, Austria; 3 Department of Internal Medicine V – Hematology and Oncology, Medical University of Innsbruck, Anichstrasse 35, 6020 Innsbruck, Austria; 4 Department of Internal Medicine III – Cardiology and Angiology, Medical University of Innsbruck, Anichstrasse 35, 6020 Innsbruck, Austria; 5 Department of Internal Medicine I – Gastroenterology, Hepatology and Endocrinology, Medical University of Innsbruck, Anichstrasse 35, 6020 Innsbruck, Austria; 6 Department of Internal Medicine II, Medical University of Innsbruck, Anichstrasse 35, 6020 Innsbruck, Austria; 7 Vice Rectorate for Teaching and Study Affairs, Medical University of Innsbruck, Fritz-Pregl-Strasse 3, 6020 Innsbruck, Austria; 8 Institute for Medical Education (IML) - Assessment and Evaluation Unit (AAE), University of Bern, Mittelstrasse 43, CH-3012 Bern, Switzerland

**Keywords:** OSCE, Familiarization, Elective course, Performance, Medical students, Active participation, Passive lecture

## Abstract

**Background:**

Assessments, such as summative structured examinations, aim to verify whether students have acquired the necessary competencies. It is important to familiarize students with the examination format prior to the assessment to ensure that true competency is measured. However, it is unclear whether students can demonstrate their true potential or possibly perform less effectively due to the unfamiliar examination format. Hence, we questioned whether a 10-min active familiarization in the form of simulation improved medical students´ OSCE performance. Next, we wanted to elucidate whether the effect depends on whether the familiarization procedure is active or passive.

**Methods:**

We implemented an intervention consisting of a 10-min active simulation to prepare the students for the OSCE setting. We compared the impact of this intervention on performance to no intervention in 5th-year medical students (*n* = 1284) from 2018 until 2022. Recently, a passive lecture, in which the OSCE setting is explained without active participation of the students, was introduced as a comparator group. Students who participated in neither the intervention nor the passive lecture group formed the control group. The OSCE performance between the groups and the impact of gender was assessed using X^2^, nonparametric tests and regression analysis (total *n* = 362).

**Results:**

We found that active familiarization of students (*n* = 188) yields significantly better performance compared to the passive comparator (Cohen´s d = 0.857, *p* < 0.001, *n* = 52) and control group (Cohen´s d = 0.473, *p* < 0.001, *n* = 122). In multivariate regression analysis, active intervention remained the only significant variable with a 2.945-fold increase in the probability of passing the exam (*p* = 0.018).

**Conclusions:**

A short 10-min active intervention to familiarize students with the OSCE setting significantly improved student performance. We suggest that curricula should include simulations on the exam setting in addition to courses that increase knowledge or skills to mitigate the negative effect of nonfamiliarity with the OSCE exam setting on the students.

**Supplementary Information:**

The online version contains supplementary material available at 10.1186/s12909-024-05091-0.

## Background

In general, assessments have the purpose of evaluating whether students possess the necessary competencies they are expected to acquire during their academic studies [1]. In this context, it is important to establish a constructive alignment between teaching and assessment to ensure that students' learning outcomes are appropriately measured [2]. However, it is also important to provide students with the opportunity to familiarize themselves with the examination format prior to the actual assessment [3–5]. This preexposure is intended to ensure that students' true competencies are accurately assessed in the examination. This is often the case with written examinations, as self-assessments or other formative assessments allow students to adapt well to the examination format. However, with structured practical examinations such as objective structured clinical examinations (OSCEs), this is often not the case. In particular, to the best of our knowledge there is only one study showing that familiarization, carried out as a formative OSCE, improved overall pass rates in a subsequent summative OSCE [4]. Consequently, it remains unclear whether students can fully demonstrate their actual capabilities or if they might perform less effectively due to the unfamiliar examination format. By adequately preparing students and familiarizing them with the OSCE format, this potential limitation could be mitigated.

Objective structured clinical examinations (OSCEs) are widely used as a reliable and valid tool to assess the clinical skills and competencies of medical students [6–9]. Although OSCEs are a useful assessment format, they still represent an "artificial" testing situation, as is the case with all structured examinations, requiring the learners to initially acquaint themselves with the general format. It is important to emphasise that this is not a matter of memorising a checklist when talking about OSCE familiarisation. On the contrary, it involves familiarising students with the unfamiliar setting and the tasks to be performed in the examination. In the current literature, only limited data are available concerning the prevalent methods employed for familiarization of individuals with the OSCE format, along with an assessment of their efficacy. It is therefore unclear how much familiarization with the OSCE format is needed to make it work. Indeed, it has been shown that increasing familiarity with a test by showing a 15-min OSCE simulation video or implementing a mock OSCE decreases stress levels [10, 11]. However, controversial data exist regarding whether increasing familiarity has an impact on students´ performance [12–20].

It is well accepted that active engagement with learning content, as opposed to passive absorption, has a positive impact on learning. This has been shown not only for the perception of learning [21, 22] but also at the level of actual student performance in general [23] and of medical students in particular [24, 25]. However, it is not known whether the learning format with a focus on familiarizing students with the exam format will affect subsequent OSCE performance. In this context, it is important to note that the alignment of the format of familiarization and the actual format of the exam could play a pivotal role. To the best of our knowledge, this has not been addressed in the literature, nor have different formats used to familiarize learners with OSCE been compared with each other.

Therefore, this study had two aims. First, we investigated the impact of a short intervention to increase students’ familiarity with the exam setting on their OSCE performance. Second, we hypothesized that OSCE performance would be superior if students actively participated to familiarize themselves with the exam environment than if the same information was provided passively in a lecture.

## Methods

### Setting and participants

The MD (medical doctoral)-program at the Medical University of Innsbruck (MUI) in Austria comprises six years. The first five years of the studies consist of practical courses, lectures, and different seminars, such as problem-based learning. Most of the time, students work with patients but solely under direct supervision. In the last year, which is called the “clinical practical year” (CPJ), students work with patients under indirect supervision. Because of that, the university established a CPJ-OSCE at the end of the 5th study year. This is a high-stakes examination designed to demonstrate that students have sufficient competence to work safely with patients. This CPJ-OSCE consists of 8 stations covering 11 different specialties (e.g., internal medicine, surgery, radiology, paediatrics, etc.). Supplemental Table 1 summarizes the main activities of the OSCE tasks of all specialities.

### Intervention

To address the first research question, the CPJ-OSCE performance of students who participated in an exam familiarization intervention was compared with that of students who did not participate in the intervention. Since 2018, MUI has established an elective course to help students become familiar with the OSCE setting. The focus of this intervention was not to foster clinical knowledge but to increase familiarity with the exam format. For this purpose, an internal medicine (IM) case similar to the cases in the summative exam was selected, and each student in the elective course was given the opportunity to once actively role-play as a candidate (10 min with subsequent feedback from faculty). It is important to note, that the course focuses on the activities of the students (e.g. taking anamnesis or providing a structured patient handover). Checklists that are used for the OSCE are neither used nor discussed within the elective course. Typically, just over half of the student population enrols in this course (= intervention group). Students who did not enrol had no prior exposure to the exam format and formed the control group. We retrospectively analysed the CPJ-OSCE performance of the two student populations in the 2018–22 cohorts and referred to them as the “discovery cohort”.

**Fig. 1:**
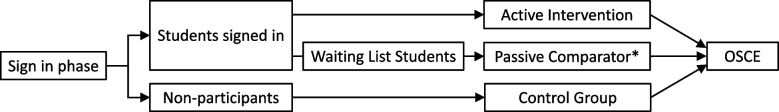
Overview—intervention, comparator, and control group. Legend: Fig. 1 depicts how the students were allocated to the respective groups (active intervention, passive comparator, and control group). In the 2018 – 2022 cohorts only two groups (active intervention and control group) exist. * The passive comparator group, consisting of students on the waiting list for the elective course, was introduced in the 2023 cohort, which is marked with an asterisk

To test our hypothesis that OSCE performance depends on the learning format of the familiarization intervention (active = role-playing of the student as a candidate vs. passive = mere transmission of information through lecture), we used a so-called comparison group in addition to the intervention and control groups in 2023. Students in the comparator group were offered the opportunity to watch a video of an IM OSCE station, and the faculty discussed afterwards some salient aspects of the OSCE format with these students. Students who registered for the elective course but were not able to attend due to the limited number of places available (students on the waiting list) were invited to attend this lecture and formed the comparator group (Fig. 1). Focus of the input was on desired student activity (e.g. anamnesis taking). OSCE checklists were neither part of the information provided nor discussed. Of note, students in the 2023 cohort who registered for the elective course and went through the active familiarization intervention (role-playing as a candidate) formed the intervention group; students who did not register for the elective course and hence did not participate in either the active or passive familiarization intervention formed the control group. The study with the 2023 cohort is referred to as the “validation cohort”.

### Outcomes

Outcome measures were OSCE performance in total and specifically in the internal medicine station as measured using the score (%) and pass/fail rates as measured by comparing the actual score to the cut-score, which is calculated using the Borderline-Group Method [26–28]. Gender (male/female) was also collected as a possible cofounding variable.

### Data analysis

Statistical analysis was conducted using SPSS software version 29.0. (SPSS Inc., Chicago, USA). Descriptive statistics of both cohorts are presented as means, medians, standard deviations (SDs) and numbers and percentages. Differences between two or more continuous and Gaussian distributed variables (OSCE scores) were calculated using Student´s t test or 1-way ANOVA. Effect sizes are stated by using either Cohen´s d or Eta^2^ and the 95% confidence interval. For comparison of noncontinuous variables (gender, pass/fail rates), the chi-square (X^2^) test was used. To assess the effect of variables such as gender or intervention group on continuous variables, we applied linear regression analysis, whereas logistic regression was applied for pass/fail rates. Multivariate adjusted models were calculated as outlined in the results section.

## Results

### Discovery cohort—2018–2022

The discovery cohort (cohorts 2018–22) consisted of 1,284 5th year medical students, of whom 687 (53.50%) were female. A total of 757 (58.96%) students participated in the elective course. Of those, 451 (59.58%) students were female.

The total OSCE score was significantly higher in participants compared to nonparticipants (707.97 vs. 694.74%, *p* < 0.001, Cohen´s d 0.307, 95%-CI 0.191–0.423). Of note, the cumulative OSCE score is a sum score of 8 OSCE stations and therefore has a maximum value of 800 percent. Pass/fail rates were very low and did not differ significantly between the two groups (see Table 1).

**Table 1:** Outcome measures in the discovery cohort—intervention vs. control – total OSCE performance

	**Intervention (*N* = 757)**			**Control (*N* = 527)**			***p***
	**Mean**	**SD**	**N/%**	**Mean**	**SD**	**N/%**	
**Total OSCE score [%]**	707.97	41.66	-	694.74	44.88	-	< 0.001^*^
**Fail rate**			5/0.6			1/0.2	0.241
**PASS rate**			752/99.4			526/99.8	0.241

Concerning the outcome measures in the internal medicine (IM) station, participants had significantly higher OSCE scores and lower fail rates. In particular, the mean scores were 95.13 and 91.95 (Cohen´s d = 0.409 (95% CI 0.296–0.521)), whereas the fail rates were 8.85% and 15.94% for participants and nonparticipants (control), respectively (Table 2).

**Table 2:** Outcome measures in the discovery cohort—intervention vs. control – OSCE station on internal medicine

	**Intervention (*N* = 757)**			**Control (*N* = 527)**			***p***
	**Mean**	**SD**	**N/%**	**Mean**	**SD**	**N/%**	
**OSCE score [%]**	95.13	7.07	-	91.95	8.68	-	< 0.001^*^
**Fail rate**			67/8.85			84/15.94	< 0.001^*^
**PASS rate**			690/91.15			443/84.06	< 0.001^*^

As a next step, we calculated the impact of the intervention and gender on IM-OSCE station scores and IM-OSCE station pass rates. As shown in Table 3, both variables had a significant impact on the pass/fail rate. Participation in the elective course almost doubled the likelihood of passing the IM station, whereas male gender lowered the chance of passing. In a multivariate Cox regression, only the intervention also had a significant impact.

**Table 3:** Logistic regression for IM-station pass rates

	**Univariate Analysis**			**Multivariate Analysis **^a^		
**Factor**	HR	95%-CI	*p*	HR	95%-CI	*p*
INTERVENTION	1.953	1.386–2.750	< 0.001^*^	1.872^a^	1.324–2.646^a^	< 0.001^*^
MALE GENDER	0.680	0.484–0.957	0.027^*^	0.744^a^	0.526–1.051^a^	0.094

*HR* hazard ratio, *95%-CI* 95% confidence interval

^a^multivariate model adjusted for the variables intervention and male gender

Similar results were obtained using linear regression, and the IM-station OSCE scores as Exp(B) for intervention and male gender were 3.176 and -1.149, respectively. In the multivariate regression, again, only the intervention remained a significant variable (Table 4).

**Table 4:** Linear regression for IM-station OSCE scores

	**Univariate Analysis**			**Multivariate Analysis **^a^		
**Factor**	HR	95%-CI	*p*	HR	95%-CI	*p*
INTERVENTION	3.176	2.311–4.041	< 0.001^*^	3.072^a^	2.198–3.945	< 0.001^*a^
MALE GENDER	-1.149	-2.017—-0.281	0.009^*^	-0.707^a^	-1.568–0.154	0.108^a^

*HR* hazard ratio, *95%-CI* 95% confidence interval

^a^multivariate model adjusted for the variables intervention and male gender

In conclusion, in the discovery cohort, we found a significant benefit for students who participated in the elective course (the intervention group) compared to nonparticipants concerning OSCE total scores, IM-station OSCE scores and IM-station pass/fail rates.

### Validation cohort—Year 2023

The validation cohort consisted of 362 5th-year medical students, of whom 188 actively participated in the elective course (= intervention) and 52 actively participated in the passive frontal lecture (= comparator). A total of 122 students were nonparticipants and formed the control group. Again, female students represented a greater proportion in the intervention and comparator groups than in the control group (see Table 5).

**Table 5:** Composition of the validation cohort (2023)

	**Intervention [n/%]**	**Comparator [n/%]**	**Control [n/%]**	**Total [n/%]**
n	188/51.93	52/14.36	122/33.70	362/100
Female	138/73.40	28/53.85	45/36.89	211/58.29

The total OSCE score was significantly higher in the intervention group than in the comparator group and nonparticipants (715.12 vs. 700.50 vs. 688.16%, *p* = 0.001, 1-way ANOVA, Eta^2^ = 0.062, 95% CI 0.02–0.112). Using Student´s t test, the difference in the total OSCE score between the control and comparator groups was not significant (*p* = 0.187), whereas we found significantly lower scores in both the control (*p* < 0.001, Cohen´s d = 0.563, 95% CI 0.330–0.795) and comparator groups (*p* = 0.025, Cohen´s d = 0.352, 95% CI 0.043–0.661) than in the intervention group. The pass rates were 100% in the intervention and comparator groups and 95.9% in the controls (X^2^, *p* = 0.007).

For the IM station, as shown in Table 6, students in the intervention group had significantly better OSCE scores and pass/fail rates. The mean scores were 92.73, 85.82 and 88.44 for the active intervention, passive comparator and control groups (1-way ANOVA, Eta^2^ = 0.076, 95% CI 0.030–0.130); the pass rates were 95.21, 82.69 and 86.89, respectively.

**Table 6:** IM-station OSCE outcome measures in the validation cohort

	**Intervention (*N* = 188)**		**Comparator (*N* = 52)**		**Control (*N* = 122)**		***p***
	**Mean/SD**	**N/%**	**Mean/SD**	**N/%**	**Mean/SD**	**N/%**	
**OSCE score [%]**	92.73/7.18		85.82/10.68		88.44/11.41		< 0.001^*^
**Fail rate**		9/4.79		9/17.31		16/13.11	0.005^*^
**PASS rate**		179/95.21		43/82.69		106/86.89	0.005^*^

We additionally calculated p values for comparisons of IM-station OSCE scores between two groups using Student´s t test. We found no significant difference between the control and comparator groups (*p* = 0.160). However, the differences between comparator and intervention (*p* < 0.001, Cohen´s d = 0.857, 95% CI 0.540–1.173) as well as between control and intervention (*p* < 0.001, Cohen´s d = 0.473, 95% CI 0.242–0.703) were significant. Furthermore, we performed additional X^2^ tests for comparisons of two groups. We found no difference in the pass/fail rates between the comparator and control (*p* = 0.47), whereas the comparisons between the active intervention and both the passive comparator (*p* = 0.002) and control (*p* = 0.02) were significant.

As in the discovery cohort, we calculated the impact of the group allocation and gender on IM-OSCE scores and pass/fail rates using linear and logistic regression analysis. The active intervention was associated with a significant and threefold increased likelihood of passing the OSCE. Male gender and the comparator group were associated with a lower chance of passing (Table 7), although both differences did not reach significant levels. In the multivariate model adjusted for group allocation and gender, only the intervention remained a significant predictor for passing the IM-station OSCE.

**Table 7:** Logistic regression for IM-station pass rates in the validation cohort

	**Univariate Analysis**			**Multivariate Analysis**		
**Factor**	HR	95%-CI	*p*	HR	95%-CI	*p*
INTERVENTION	3.002	1.282–7.033	0.011^*^	2.945	1.204–7.205	0.018^*^
COMPARATOR	0.721	0.296–1-757	0.472	0.715	0.291–1.757	0.464
MALE GENDER	0.691	0.34–1.401	0.305	0.949	0.447–2.016	0.892

*HR* hazard ratio, *95%-CI* 95% confidence interval. The multivariate model is adjusted for the variables intervention, comparator and male gender

Concerning the IM-station OSCE score, the variables intervention and male gender showed significant positive and negative associations in the univariate linear regression, respectively (Table 8). In a multivariate model adjusted for intervention, comparator and male gender again, only the intervention remained a significant predictor for higher IM-station OSCE scores.

**Table 8:** Linear regression for IM-station OSCE score in the validation cohort

	**Univariate Analysis**			**Multivariate Analysis**		
**Factor**	HR	95%-CI	*p*	HR	95%-CI	*p*
INTERVENTION	4.292	2.161–6.423	< 0.001^*^	4.025	1.761–6.288	0.001^*^
COMPARATOR	-2.615	-5.651- 0.421	0.091	-2.739	-5.798–0.319	0.079
MALE GENDER	-2.103	-4.122- -0.085	0.041^*^	-0.732	-2.809–1.345	0.489

*HR* hazard ratio, *95%-CI* 95% confidence interval. The multivariate model is adjusted for the variables intervention, comparator and male gender

In summary, we found that in the validation cohort, the introduction of an elective course, where students actively participated in role playing as candidates, had significant beneficial effects on students’ OSCE performance. There was no significant effect of the comparator, a passive frontal lecture, on the same outcome measures.

## Discussion

Our results suggest that familiarity with the exam setting has a beneficial effect on examinees’ performance in a summative OSCE in undergraduate medical education. In addition, our data show that an intervention to increase familiarity with the OSCE examination format cannot be achieved by means of a passive transfer of information but must include an active component that is aligned with the summative examination format. In this context, it is important to stress the fact that in neither intervention (active or passive) any OSCE checklist items were used by the faculty nor discussed with the students. Only the desired clinical activities within the OSCE tasks (e.g. taking a history or suggesting additional diagnostic tests) were practised (intervention) respectively discussed with the students (comparator).

We found that students who participated in the 10-min familiarization intervention had significantly higher OSCE scores (total and internal medicine) and lower failure rates in the internal medicine station compared to nonparticipants. There is ample evidence that simulation training improves clinical skills [29, 30], but controversial data also exist. In particular, a peer-assisted mock OSCE, in which students complete an entire 10-station OSCE, had no significant effect on the actual OSCE performances compared to nonparticipants [19]. However, in this study, the obvious aim was to increase knowledge or skills. Additionally, it is not clear if the peers had enough knowledge of the actual OSCE to prepare the students. It is important to note that our short intervention may not have a significant beneficial effect on the students’ actual clinical knowledge or skills. Indeed, the main purpose of our intervention was to specifically increase familiarity with the exam setting. This is clearly demonstrated by the fact that our short familiarization intervention, carried out on an IM case, had a positive impact on performing in OSCE cases involving completely different specialties, as measured via the total OSCE score. Moreover, the difference in performance between the two student groups (intervention and control) was statistically significant and also had a moderate effect (Cohen´s d = 0.409). That is, from our perspective, higher than we would have estimated for a 10-min intervention.

More interestingly, our study also suggests how an exam familiarization intervention should be designed to help improve subsequent OSCE performance. In general, students learn more when they are actively engaged than they do in a passive lecture environment [31]. Kooloos et al. have shown that active learning methods may contribute to higher achievement in terms of summative test scores [32]. These findings are in line with our results: students in the familiarization intervention with active participation outperformed their peers who only received passive information about the exam format. The fact that showing a video in the OSCE format does not have an impact on subsequent performance in the OSCE has also been demonstrated by others. A 15-min video intended to increase familiarity with the exam format decreased stress levels in nursing students but was not accompanied by an increase in OSCE scores [10]. An intriguing explanation for this finding is provided by Deslaurier et al. They concluded that by mere passive absorption of information but not taking part in the activity, the students felt overconfident and did not prepare themselves sufficiently for the actual OSCE. [35]. It seems that not only is active engagement on the part of the students important but also the alignment of the familiarization activity with the actual OSCE format seems to be crucial. In a study by Aster et al., students' use of serious games with virtual patients did not have an effect on performance in a subsequent OSCE [25]. In our study, where the familiarization activity and the OSCE format were aligned, a 10-min intervention improved the subsequent performance on the OSCE.

In univariate regression analysis, male gender was a significant predictor of higher failure rates and lower OSCE scores. Similar results were published by Haist et al. [33], who showed that young women outperform young men in a clinically based performance examination and were less likely to experience academic difficulty. This is also in line with Brand et al., who showed that females scored significantly higher than males among 3rd dental students [14]. Gorth et al. published that women receive higher scores in the majority of clinical performance [34], and Komasawa et al. found significantly higher scores and performance in integrative tests and clinical clerkships of female compared to male students [35]. Of note, there are also controversial published data showing no difference in academic performance between female and male medical students [36]. In this context, it is important to note that the gender effect in our study became not statistically significant after adjusting for our intervention.

This study has several limitations. The primary constraint of this study pertains to the nonrandom assignment of students to their respective groups. It is plausible that students who choose to participate in an elective course possess certain characteristics that set them apart from students who do not opt for such a course. Consequently, one could argue that motivated students who enroll in our intervention inherently demonstrate superior performance compared to students who do not enroll in the course. Therefore, it would be the distinct characteristics of the two student populations, rather than the intervention itself, that would account for the observed superior performance in our study. Hence, it was crucial to include a comparator group in the 2023 cohort. The students in the comparator group also voluntarily enrolled in the course; however, they were assigned to the comparator due to capacity limitations. The disparity in performance between the intervention group and the comparator group identified in our study can thus be attributed solely to the intervention. Furthermore, the absence of performance differences between the control and comparator groups suggests that there is no systematic distinction between students who enrolled in the course and those who did not. Despite our efforts to mitigate this issue by introducing a comparator group for motivated students, it is evident that this does not constitute a randomized allocation. Another limitation is the unequal distribution of students across groups. However, it was not permissible to randomize and distribute students evenly, as the intervention is a routinely held elective course in our faculty.

Our data suggest that familiarization with the exam format is crucial to achieve optimal performance during the examination. Therefore, all students should be given the opportunity to undergo such familiarization before they are required to take a similar examination for the first time. Our study further suggests that the familiarization process should align with the nature of the examination. In the case of a practical examination, familiarization should also involve active participation, as a passive transfer of information alone does not appear to be effective in familiarizing students with subsequent performance assessment.

Our results should be verified in a randomized study. Additionally, we believe that clarification studies are warranted to gain a deeper understanding of the key elements of familiarization with the assessment format but not content that is responsible for optimizing performance.

## Conclusion

In conclusion, in our study, a short 10-min active intervention to increase familiarity with the exam setting yielded significantly better performance in 5th-year medical students in a summative OSCE. Our findings suggest that the active participation of the students and an alignment between the familiarization intervention and the subsequent OSCE format are crucial to achieving this result.

### Supplementary Information

**Additional file 1:** **Supplemental Table 1.** OSCE subspecialties and main checklist items.

## Data Availability

The datasets used and/or analysed during the current study are available from the corresponding author upon reasonable request.

## Abbreviations

CPJ: Clinical practical year
ENT: Ear, nose, throat
IM: Internal medicine
MUI: Medical University of Innsbruck
OSCE: Objective Structured Clinical Examination
SD: Standard deviation
