# Supplementary material for: Impact of familiarity with the format of the exam on performance in the OSCE of undergraduate medical students – an interventional study
Source: BMC Med Educ. 2024 Feb 23;24:179. doi: 10.1186/s12909-024-05091-0 (PMC10893607; doi:10.1186/s12909-024-05091-0)
Supplement: Supplementary file 1 — Additional file 1: Supplemental Table 1. OSCE subspecialties and main checklist items. [file 12909_2024_5091_MOESM1_ESM.docx]

Supplemental Table 1. OSCE subspecialties and main checklist items.

|  | **Specialities** | | | | | | | | | | |
| --- | --- | --- | --- | --- | --- | --- | --- | --- | --- | --- | --- |
| **Main items** | Clinical Pharmacology | Emergency Medicine | ENT | Gynecology | Internal Medicine | Neurology | Ophthalmology | Pediatrics | Psychiatry | Radiology | Surgery |
| Introduction | X | X | X |  | X | X | X | X | X |  | X |
| Anamnesis | X | X | X |  | X | X | X | X | X |  | X |
| Diagnostic Measures and Interpretation | X | X | X | X | X | X | X | X |  |  | X |
| Structured Interpretation of medical imaging |  |  |  | X |  |  | X |  |  | X |  |
| Therapeutic Measures | X | X |  | X | X |  |  | X |  |  | X |
| Differential Diagnosis |  | X | X | X | X | X |  | X | X | X | X |
| Professionality (verbal, non-verbal) | X | X | X | X | X | X | X | X | X |  |  |
| Communication | X | X | X | X | X | X | X | X | X |  |  |
| Empathy | X | X | X | X | X | X | X | X | X |  |  |
| Patient Handover | X | X | X | X | X | X |  | X | X |  | X |

ENT … ear, nose, throat. Note, actual checklist items may be different from speciality to speciality. For example, diagnostic measures in internal medicine may include an echocardiography and electrocardiogram whereas in surgery computed tomography or ultrasound imaging are the right measures.
